# Infantile hepatic hemangioma and hepatic mesenchymal hamartoma in an infant associated with placental mesenchymal dysplasia: a case report

**DOI:** 10.1186/s40792-022-01519-1

**Published:** 2022-08-29

**Authors:** Shunsuke Fujii, Kyoko Mochizuki, Hidehito Usui, Norihiko Kitagawa, Sayoko Umemoto, Mio Tanaka, Yukichi Tanaka, Masako Otani, Kumiko Nozawa, Kenji Kurosawa, Masayo Kagami, Masato Shinkai

**Affiliations:** 1 Department of Surgery, Kanagawa Children’s Medical Center, 2-138-4, Mutsukawa, Minami-Ku, Yokohama, Japan; 2 Department of Pathology, Kanagawa Children’s Medical Center, 2-138-4, Mutsukawa, Minami-Ku, Yokohama, Japan; 3 Department of Diagnostic Pathology, Yokohama City University Medical Center, 4-57, Urafune-Cho, Minami-Ku, Yokohama, Japan; 4 Department of Radiology, Kanagawa Children’s Medical Center, 2-138-4, Mutsukawa, Minami-Ku, Yokohama, Japan; 5 Department of Medical Genetics, Kanagawa Children’s Medical Center, 2-138-4, Mutsukawa, Minami-Ku, Yokohama, Japan; 6 Department of Molecular Endocrinology, National Research Institute for Child Health and Development, 2-10-1, Okura, Setagaya-Ku, Tokyo, Japan

**Keywords:** Hepatic infantile hemangioma, Hepatic mesenchymal hamartoma, Placental mesenchymal dysplasia, Propranolol, Tumorectomy

## Abstract

**Background:**

Although infantile hepatic hemangioma and hepatic mesenchymal hamartoma are relatively common in benign pediatric liver tumors, coexistence of the two tumors is rare. Placental mesenchymal dysplasia is also a rare disorder. We report the case of a baby girl born after a pregnancy complicated by placental mesenchymal dysplasia, who developed both infantile hepatic hemangioma and hepatic mesenchymal hamartoma.

**Case presentation:**

The patient was born at 32 weeks and 5 days of gestation for impending placental abruption, weighing 1450 g. Liver tumors, composed of both hypervascular solid and large cystic lesions, were detected after birth and markedly increased to create abdominal distention within 9 months. Diagnostic imaging suspected the coexistence of infantile hepatic hemangioma and cystic hepatic mesenchymal hamartoma. Following propranolol therapy for infantile hepatic hemangioma and needle puncture of a large cyst, the cystic lesions and adjacent hypervascular lesions were partially resected via laparotomy. Pathological findings confirmed the coexistence of hepatic mesenchymal hamartoma and infantile hepatic hemangioma, which had no association with androgenetic/biparental mosaicism. The postoperative course was uneventful, and the tumor had not regrown after 3 years.

**Conclusions:**

Although the coexistence of infantile hepatic hemangioma and hepatic mesenchymal hamartoma associated with placental mesenchymal dysplasia is extremely rare, the pathological and pathogenetic similarities between these disorders suggest that they could have derived from similar embryologic origins rather than being a mere coincidence. Further follow-up is required, with careful attention to the potential for malignant hepatic mesenchymal hamartoma transformation.

## Background

Liver tumors comprise only 1–2% of all pediatric tumors, and one-third are benign [1]. Infantile hepatic hemangioma (IHH) is the most common benign liver tumor, followed by hepatic mesenchymal hamartoma (HMH). IHH and HMH account for 12% and 8% of all pediatric liver tumors, respectively [1]. Histologically, infantile hemangioma is a proliferative lesion of the vascular endothelium that is positive for glucose transporter 1 (GLUT-1) on immunostaining [2]. HMH is characterized by an admixture of epithelial components, such as bile ducts and mesenchymal connective tissue, and serum alpha-fetoprotein (AFP) is moderately elevated in some cases [3]. The coexistence of IHH and HMH is rare [4–7].

Placental mesenchymal dysplasia (PMD) is a rare disorder characterized by placentomegaly, dilatation of chorionic vessels, and areas of villous cystic changes [8]. The incidence is 0.02% of all pregnancies. Though it resembles a partial hydatidiform mole, PMD may coexist with a normal fetus. Fetuses associated with PMD are at risk of intrauterine growth restriction and fetal or neonatal death. There are reports of an association between PMD and HMH [8–11]; however, the coexistence of IHH and HMH associated with PMD has never been reported. Herein, we report the case of an infant with coexisting IHH and HMH complicated with maternal PMD, and we discuss the association between and appropriate treatment for these diseases.

## Case presentation

**Fig. 1:**
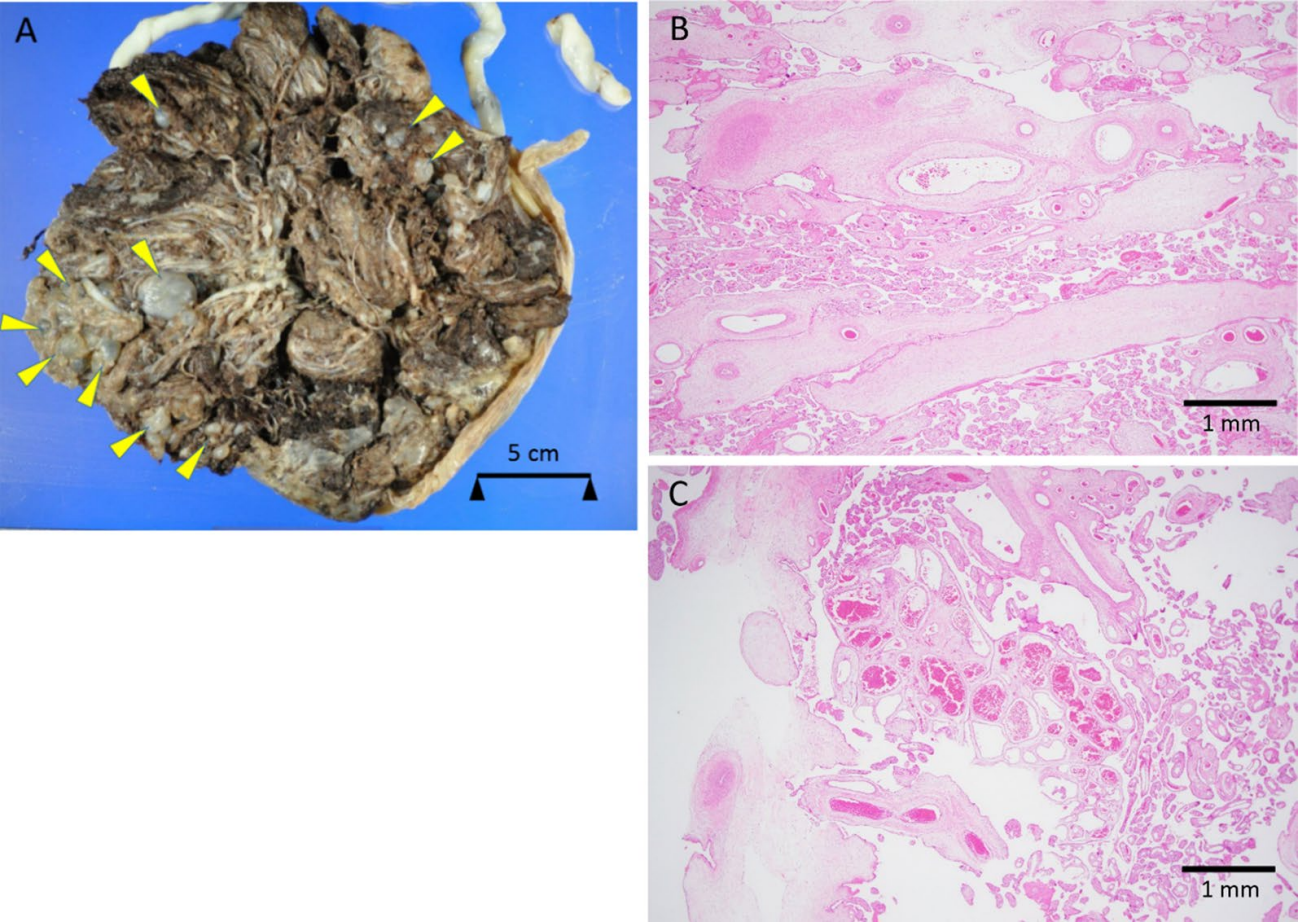
**A** Maternal surface of placenta shows multiple cysts of various size (arrowheads). B Microscopically, the placenta shows enlarged stem villi with hydropic changes (hematoxylin and eosin). **C** Hemangioma-like collections of abnormal blood vessels are slightly visible (hematoxylin and eosin)

A healthy 30-year-old gravida 1, para 1 woman was diagnosed with PMD at 15 weeks of gestation with prenatal fetal ultrasound (US); her pregnancy was otherwise uneventful. At 32 weeks and 5 days of gestation, emergency cesarean section was performed for impending placental abruption. A baby girl was born with a birth weight of 1450 g and Apgar scores of 8 and 9 at 1 and 5 min, respectively. The delivered placenta was enlarged (23.6 × 23.5 × 6.5 cm) and heavy (1209 g), and the maternal surface had multiple cystic villi (Fig. 1A). Microscopically, the placenta included hydropic stem villi containing dilated vessels surrounded by loose mesenchymal tissue that were admixed with normal villi (Fig. 1B). Trophoblast proliferation was not observed. These findings are consistent with those of PMD. A hemangioma-like aggregation of abnormal blood vessels was observed (Fig. 1C). The baby girl was admitted to the neonatal intensive care unit because of transient tachypnea and very low birth weight. Abdominal US showed that she had multiple 2-cm-sized cystic tumors in both the right and left lobes of the liver (no obvious solid tumors were identified). The infant had no external malformations. She was discharged at 50 days of age, without any complications.

**Fig. 2:**
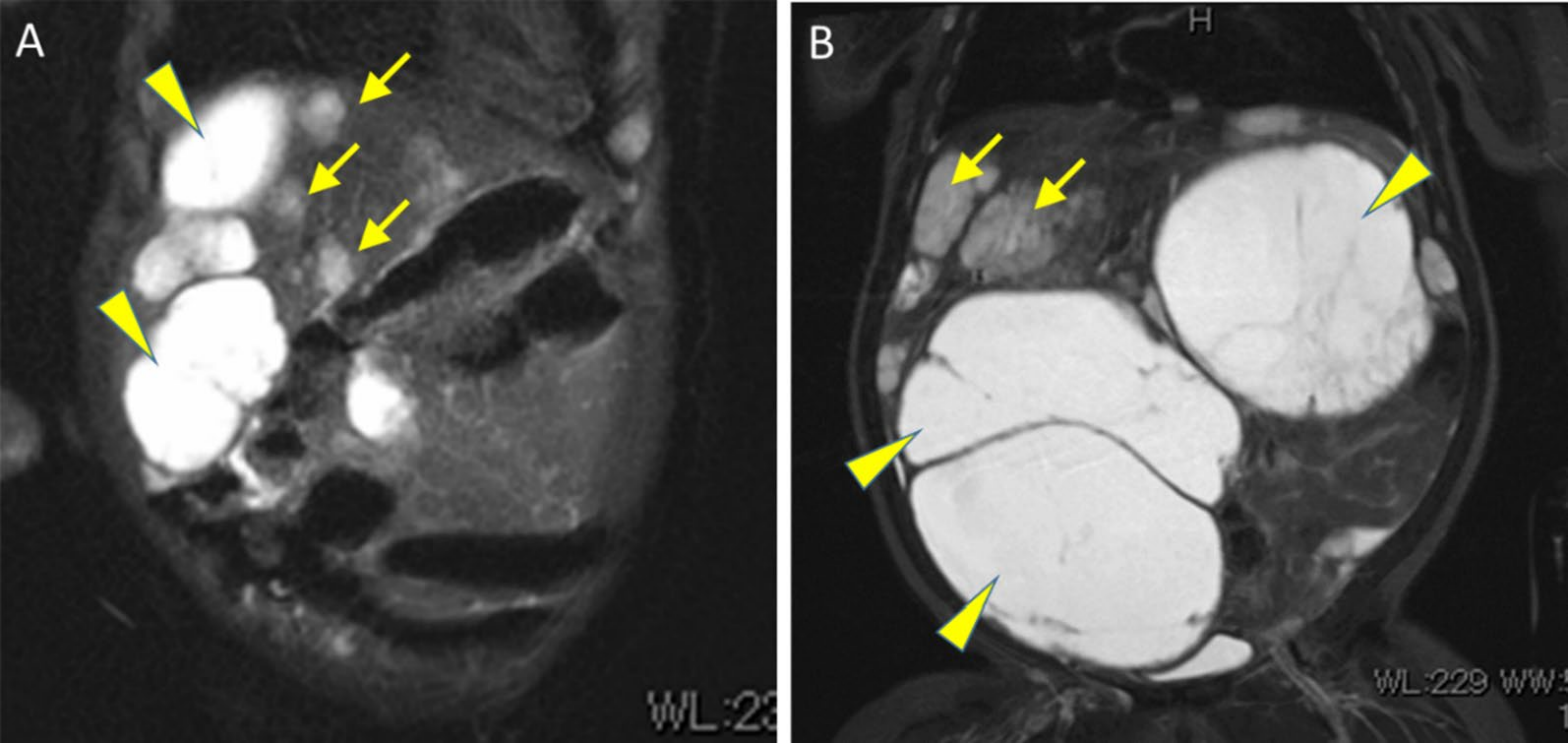
Coronal T2-weighted MR image at 2 months (**A**) and at 9 months (**B**). Both cystic (arrowheads) and solid (arrows) components show rapid increases after 7 months

At 2 months of age, she was referred to our institution for evaluation and management of liver tumors. Magnetic resonance imaging (MRI) revealed coexisting multifocal cystic and solid components in both hepatic lobes (Fig. 2A). The cystic components, 2–4 cm in diameter, showed high signal intensity on T2-weighted images and low signal intensity on T1-weighted images. The solid components, sized several millimeters to 1 cm in diameter, had high signal intensity on T2-weighted images, and even lower signal intensity than cystic lesions on T1-weighted images. Since the lesions were considered benign tumors, such as HMH and IHH, she underwent a regular follow-up as an outpatient. At 9 months, she was hospitalized for progressive abdominal distention and poor oral intake. AFP was slightly elevated for her age (350.9 ng/ml). MRI showed marked enlargement of both cystic and solid components of the liver tumors, which now occupied the abdominal cavity (Fig. 2B). Contrast-enhanced computed tomography showed early peripheral enhancement with delayed progression to the center of the solid components. These findings further supported the previous suspicions that the cystic and solid components were HMH and IHH, respectively.

The largest cyst was punctured percutaneously and aspirated to relieve abdominal distention, and oral propranolol (2 mg/kg/day) was started to promote regression of the hemangioma component. Her clinical condition temporarily improved and the volume of some hemangioma components was reduced, however; the propranolol effect was limited, and the cystic lesion returned to its original size. Laparotomy was performed at age 10 months. During the procedure, the two largest cystic tumors, located in the right and left hepatic lobes, respectively, were excised along with their adjacent small solid tumors. Postoperative recovery was uneventful, and propranolol treatment was continued until 1 year of age. At the age of 4 years, the patient was doing well, and follow-up MRI showed no HMH regrowth and a trend to decreasing IHH volume.

**Fig. 3:**
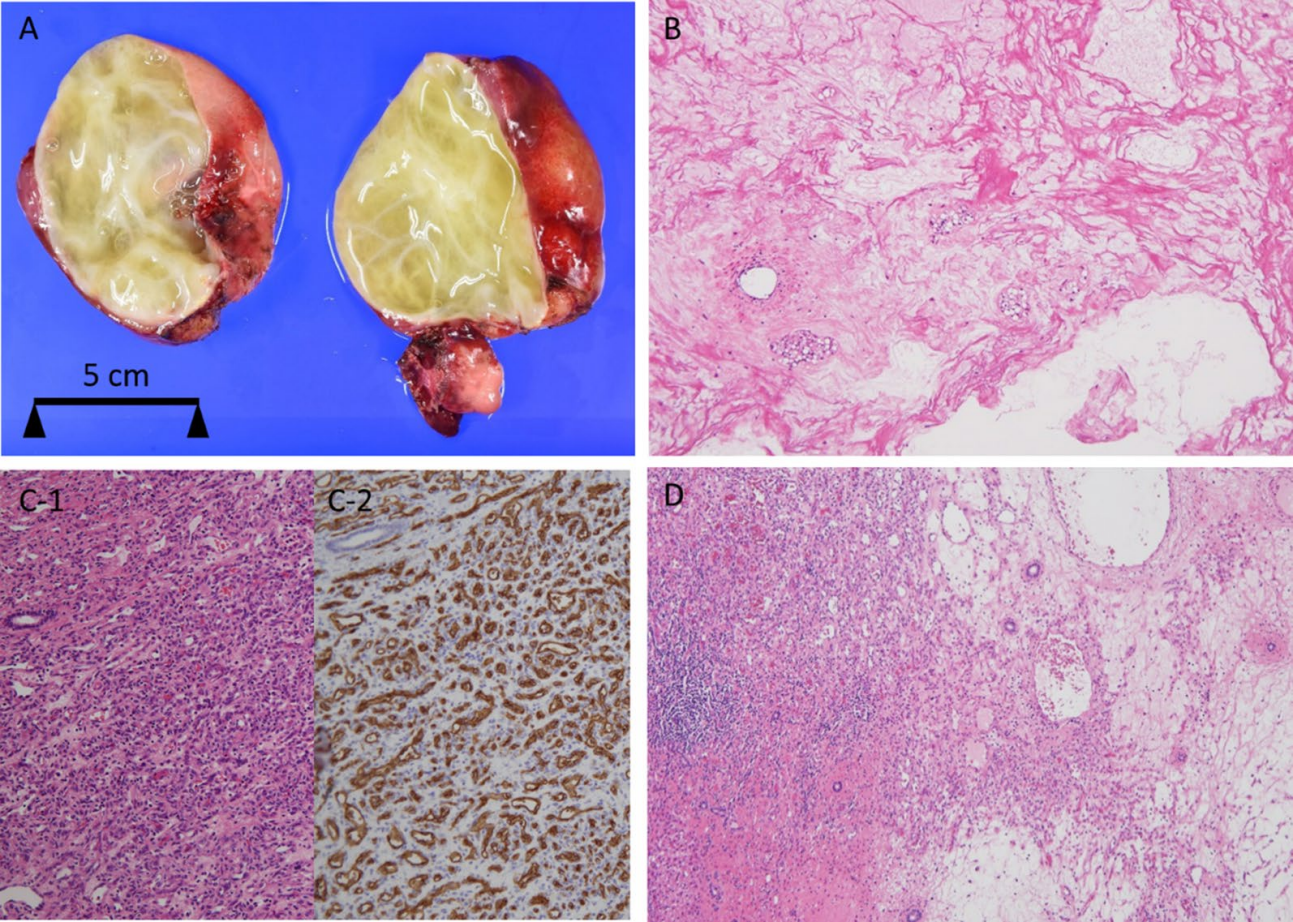
**A** Cut section of the cystic lesion in the left lobe revealing spongy, white, and solid components with mucinous material within. **B** Microscopically, spongy white lesions show loose mesenchymal component with myxoid material, scattered fibroblasts and vessels (hematoxylin and eosin, original magnification × 40). **C-1** Brownish nodule in non-cystic area showing proliferation of capillaries and small vessels (hematoxylin and eosin, original magnification × 100). **C-2** These lesional cells are positive for GLUT-1 by immunostains. **D** Left area showing IHH and the right area, HMH; the boundary is obscure (hematoxylin and eosin, original magnification × 40)

The left lobe cyst measured 115 × 105 × 70 mm, and the right lobe cyst was 100 × 62 × 48 mm. On the cut section, both lesions contained spongy, white, and solid components with multicystic spaces that were incompletely separated by loose, fibrous septa and filled with mucinous material (Fig. 3A). Microscopically, the spongy white tissue showed a loose mesenchymal component with myxoid material, scattered fibroblasts, and no overt atypical epithelial elements of heterologous differentiation (Fig. 3B). These findings are suggestive of HMH. In addition, brownish nodular lesions were observed in the non-cystic area of the left lobe. These showed proliferation of capillaries and small vessels, the lining cells of which were positive for CD34 and GLUT-1 (Fig. 3C-1, 2). These findings are consistent with IHH. The boundary between the HMH and IHH was ambiguous (Fig. 3D). Methylation analysis with pyrosequencing using genomic DNA from HMH, IHH, and normal liver tissue revealed a normal methylation status in nine differentially methylated regions (H19, PEG1, PEG10, Kv, IG, MEG3, SNRPN, ZAC1, GNAS A/B), which are involved in the development of known imprinting disorders; therefore androgenetic/biparental mosaicism (ABM) is not involved in these tumors and the normal liver tissue [12].

## Discussion

The coexistence of IHH and HMH is rare, and there have been no reports of PMD associated with these two pathologies. The patient described in our report was well for some time after birth and received regular follow-up. However, at the age of 9 months, the rapid growth of IHH and HMH caused abdominal distension and poor oral intake. The combination of propranolol therapy for IHH and partial tumorectomy for HMH resulted in extensive tumor volume reduction without post-surgical regrowth. Although the etiology of IHH, HMH, and PMD has not yet been fully elucidated, their simultaneous occurrence in our patient suggests that some common etiological mechanisms underlie the three pathologies.

It has been previously speculated that HMH could contain hemangiomatous elements. However, to the best of our knowledge, the coexistence of IHH and HMH has been reported only six times in the literature (Table 1) [4–7]. All patients were female. They presented with symptoms such as abdominal compartment syndrome from rapid tumor growth and required interventions including cyst puncture and tumorectomy. One patient died of heart failure despite treatment and another required liver transplantation (LT). In two cases, IHH developed after HMH resection, whereas in the other four cases, IHH and HMH occurred simultaneously. The possible common pathogenic mechanisms have not been sufficiently discussed in the literature.

**Table 1:** Reports on the coexistence of infantile hepatic hemangioma (IHH) and hepatic mesenchymal hamartoma (HMH)

Report	No. of patients	Sex/age	Associating findings	Treatment/outcome at report time
Bejarano, et al. (2003) [4]	1	Female/neonate	Hepatic and cutaneous infantile hemangioma developed late, 3 months after HMH resection	Liver transplant was performed due to respiratory compromise from rapid IHH growth. She survived
Hsiao, et al. (2007) [5]	1	Female/4 months	Progressive abdominal distension resulted in poor oral intake	Hepatic segmentectomy was performed after cyst aspiration
Behr, et al. (2012) [6]	3	Female/9 months	Patient also had cutaneous infantile hemangioma	The cyst showed regrowth and spontaneous regression after cyst aspiration
		Sex not mentioned/neonate	Hepatic and cutaneous infantile hemangioma developed after HMH resection	Cystectomy was followed by steroid therapy
		Female/neonate	Cardiac failure developed just after birth	Patient died at 11 days despite steroid therapy
Berte, et al. (2018) [7]	1	Female/1 month	A sudden abdominal compartment syndrome developed after needle biopsy	Cystectomy repeated 3 times in conjunction with steroid and beta blocker therapy
Our case (2022)	1	Female/9 months	Maternal PMD. Progressive abdominal distension resulted in poor oral intake	Cystectomy was performed in conjunction with beta blocker therapy

Moreover, our patient was also diagnosed with PMD. There have been reports of HMH associated with PMD [8–11]. HMH and PMD have similar histologies, including mesenchymal tissue growth with myxoid changes, and it is speculated that the pathogenetic origin is the same. Kitano et al. postulated that thrombin in placental vessels might embolize the fetal liver, resulting in mesenchymal hamartoma via a reactive response to ischemia [10]. They also considered that HMH could have metastasized from the PMD in utero.

More recently, it has been suggested that HMH and PMD could be caused by ABM. First, a mixed cell population consisting of normal and androgenetic (complete paternal uniparental disomy) cells was identified in the PMD placenta [13, 14]. The presence of ABM was later confirmed in HMH associated with PMD [11]. Reed, et al. have conjectured that ABM may cause PMD and HMH through at least two mechanisms. First, androgenetic cells greatly increase the likelihood of recessive traits with a genome-wide paternal uniparental disomy. Second, androgenetic cells may influence some phenotypes associated with paternal uniparental disomy through abnormal expression of paternally imprinted genes. However, ABM was not detected in our patient, and thus, other genetic factors might also be involved.

Hemangiomas (liver and skin) in neonates have been associated with PMD [14] and placental hemangiomas [15, 16]. Although the pathogenesis of infantile hemangioma is poorly understood, some researchers have hypothesized that its origin is embolization of placental chorionic villous mesenchymal core cells [2]. In addition, hypoxia seems to be involved in the development of both infantile hemangioma and HMH [17]. Impaired placental circulation due to PMD may create a hypoxic fetal environment, leading to the development of IHH and HMH.

In our patient, the combination of propranolol therapy for IHH and tumorectomy for the two largest HMH lesions diminished the progressive abdominal distension during infancy. Three years after the surgery, the HMH showed no regrowth, and the IHH was gradually regressing. Still, the optimal treatment and prognosis for our patient remain unclear. IHH is known to regress over time [2, 17] and should be expected to improve in the future. For HMH, there is a risk of malignant transformation to undifferentiated embryonal sarcoma, and complete resection is preferred [3]. However, the HMH was diffusely distributed in both hepatic lobes in our patient, and complete resection was impossible without LT. Treatment options included sequential tumorectomy with follow-up or LT. Because HMH containing-hemangioma components are considered more likely to regress [3], complete resection may become possible over time. ABM, which is often associated with PMD and HMH, was not observed in this patient. Loss of heterozygosity associated with ABM has been reported to increase the predisposition to malignancy. In such cases, pre-emptive LT may be more appropriate than follow-up [18]. For our patient, no ABM is present, and further follow-up is required with close attention to the potential for malignant HMH transformation.

## Conclusions

Although the coexistence of IHH and HMH in infants born to women with PMD is extremely rare, the pathological and pathogenetic similarities between these disorders suggest that they could share an embryological origin, rather than being mere coincidence. The treatment and prognosis of these conditions remain unclear, and careful follow-up and additional treatments should be considered.

## Data Availability

Not applicable.

## Abbreviations

IHH: Infantile hepatic hemangioma
HMH: Hepatic mesenchymal hamartoma
PMD: Placental mesenchymal dysplasia
ABM: Androgenetic/biparental mosaicism
GLUT-1: Glucose transporter 1
AFP: Alpha-fetoprotein
US: Ultrasound
MRI: Magnetic resonance imaging
LT: Liver transplantation
